# 1-year above-recommendation screen use and internalizing and externalizing behaviours in French children aged 3 to 14 years

**DOI:** 10.1192/j.eurpsy.2023.1125

**Published:** 2023-07-19

**Authors:** A. Descarpentry, C. Davisse-Paturet, C. Galera, J.-B. Hazo, M. Melchior, A. Rouquette

**Affiliations:** 1 Université Paris-Saclay, UVSQ, Inserm, CESP, Villejuif; 2 Inserm U 1219, Bordeaux Population Health Research Center; 3 Université de Bordeaux; 4 Department of Child and Adolescent Psychiatry, Centre Hospitalier Charles Perrens, Bordeaux; 5 DREES - Direction de la Recherche, des Etudes, de l’évaluation et des statistiques; 6 Inserm Sorbonne Université, IPLESP, ERES UMRS 1136, Paris; 7 Epidemiology and Public Health Service, AP-HP, Hôpitaux Universitaires Paris-Saclay, Le Kremlin-Bicêtre, France

## Abstract

**Introduction:**

The context of the COVID-19 pandemic has changed the daily life of families and children. Screen exposure was increased during this period to maintain social relationships, work remotely, and occupy leisure time.

**Objectives:**

To explore the association of continued above-recommendation screen use for one year since May 2020 with behaviour problems in the summer of 2021 in children aged 3 to 14 years.

**Methods:**

Data came from the French EpiCov cohort study, and were collected in May 2020 and at first (Autumn 2020) and second follow-up (Summer 2021) among 1,089 participants with children aged 3 to 14. Children had a 1-year above-recommendation screen use if their daily mean time exceeded recommendations at the three follow-up times (one hour, for children aged 3-5, two for the older ones). Behaviour problems were assessed using the Strengths and Difficulties Questionnaire (SDQ) and valid cut-offs for Internalizing (emotional or peer problems) and Externalizing (conduct problems or hyperactivity/inattention) problems completed in summer 2021. Data were analysed using adjusted logistic regression.

**Results:**

1-year above-recommendation screen use was not associated with internalizing problems (OR [95% CI]: 1.20 [0.90-1.59]). Regarding the subscales, it was associated with a higher risk of peer problems (1.42 [1.04-1.95]). A higher risk of externalizing problems was found only in 11-14-year-olds (1.63 [1.01-2.63]), especially conduct problems in 11-14-year-olds (1.91 [1.15-3.22]) but not in other age groups.

**Conclusions:**

This study found that maintaining screen time beyond recommendations for 1 year since the onset of the pandemic was associated with peer problems in children aged 3-14 years and externalizing and conduct problems in 11-14 years. Despite this very specific context, exposure to screens is not trivial. If this situation were to occur again, we would have to anticipate, with prevention messages, by keeping schools open.

**Disclosure of Interest:**

None Declared

